# Telling plant species apart with DNA: from barcodes to genomes

**DOI:** 10.1098/rstb.2015.0338

**Published:** 2016-09-05

**Authors:** Peter M. Hollingsworth, De-Zhu Li, Michelle van der Bank, Alex D. Twyford

**Affiliations:** 1 Royal Botanic Garden Edinburgh, 20A Inverleith Row, Edinburgh EH3 5LR, UK; 2 Germplasm Bank of Wild Species, Kunming Institute of Botany, Chinese Academy of Sciences, 132 Lanhei Road, Heilongtan, Kunming, Yunnan 650201, People's Republic of China; 3 Department of Botany and Plant Biotechnology, University of Johannesburg, Auckland park, Johannesburg PO Box 524, South Africa; 4 Ashworth Laboratories, Institute of Evolutionary Biology, University of Edinburgh, Edinburgh EH9 3FL, UK

**Keywords:** plant DNA barcoding, next-generation sequencing, genome skimming, species discrimination, hybrid baits

## Abstract

Land plants underpin a multitude of ecosystem functions, support human livelihoods and represent a critically important component of terrestrial biodiversity—yet many tens of thousands of species await discovery, and plant identification remains a substantial challenge, especially where material is juvenile, fragmented or processed. In this opinion article, we tackle two main topics. Firstly, we provide a short summary of the strengths and limitations of plant DNA barcoding for addressing these issues. Secondly, we discuss options for enhancing current plant barcodes, focusing on increasing discriminatory power via either gene capture of nuclear markers or genome skimming. The former has the advantage of establishing a defined set of target loci maximizing efficiency of sequencing effort, data storage and analysis. The challenge is developing a probe set for large numbers of nuclear markers that works over sufficient phylogenetic breadth. Genome skimming has the advantage of using existing protocols and being backward compatible with existing barcodes; and the depth of sequence coverage can be increased as sequencing costs fall. Its non-targeted nature does, however, present a major informatics challenge for upscaling to large sample sets.

This article is part of the themed issue ‘From DNA barcodes to biomes’.

## 1. Introduction

Despite centuries of taxonomic effort, the characterization of plant species diversity remains a substantial and important challenge. Although plants are undoubtedly well understood compared to mega-diverse groups like insects, recent estimates suggest that around 70 000 flowering-plant species await discovery [1]. Beyond finding new species, existing taxonomic accounts need reconciling and updating, and there is also the wider practical challenge of assigning unidentified specimens to known species. This latter point is particularly pertinent where the available material is sub-optimal (e.g. juvenile, fragmented, processed) or where available levels of taxonomic expertise are low.

DNA barcoding involves the standardized use of one or a few DNA regions to tell species apart [2]. In this paper, we summarize the extent to which DNA barcoding of plants [3] is providing practical progress to address these challenges and also explore the opportunities presented by the ongoing development of new sequencing technologies.

## 2. Standard plant barcodes

There is no single plant barcode that matches the universality and resolving power of Cytochrome Oxidase (C01) in animals [2]. Most specimen-based plant-barcoding studies use one or a few plastid regions (e.g. the protein coding ‘core barcodes' *rbcL* and *matK*, and the non-coding spacer *trnH-psbA*) and the internal transcribed spacer (ITS) regions of nuclear ribosomal DNA (ITS—either its entirety or just the ITS2 region) [4–7]. Plant studies focusing on mixed templates and/or degraded DNAs (e.g. environmental samples) typically use the P6 loop of the plastid *trnL* intron, whose short length and conserved primer sequences make it particularly amenable to amplification and short-read sequencing via next-generation sequencing (NGS) technologies [8,9].

In many animal groups, the close concordance of species with barcode sequence clusters enables the semi-automated quantification of species diversity [10,11]. However, plant-plastid and ribosomal-DNA barcodes typically have lower discriminatory power [12] and do not lead to tight clustering of conspecifics separated by clear discontinuities from other species in sequence space. Instead, there is typically a graded continuum of intra- and interspecific distances, with barcodes commonly shared among related species [12]. There are two main implications of this. Firstly, standard plant barcodes are best suited to being molecular augmentations to existing classifications, rather than having the resolving power to act in a stand-alone fashion to define a species-level framework. Secondly, in using plant barcodes, attention should be given at the outset to ensuring a match between the resolving power of the technique, and the information that is required from the study. Examples of the range of studies plant barcodes are being used for are given below.

### (a) Species discovery

**Figure 1.**
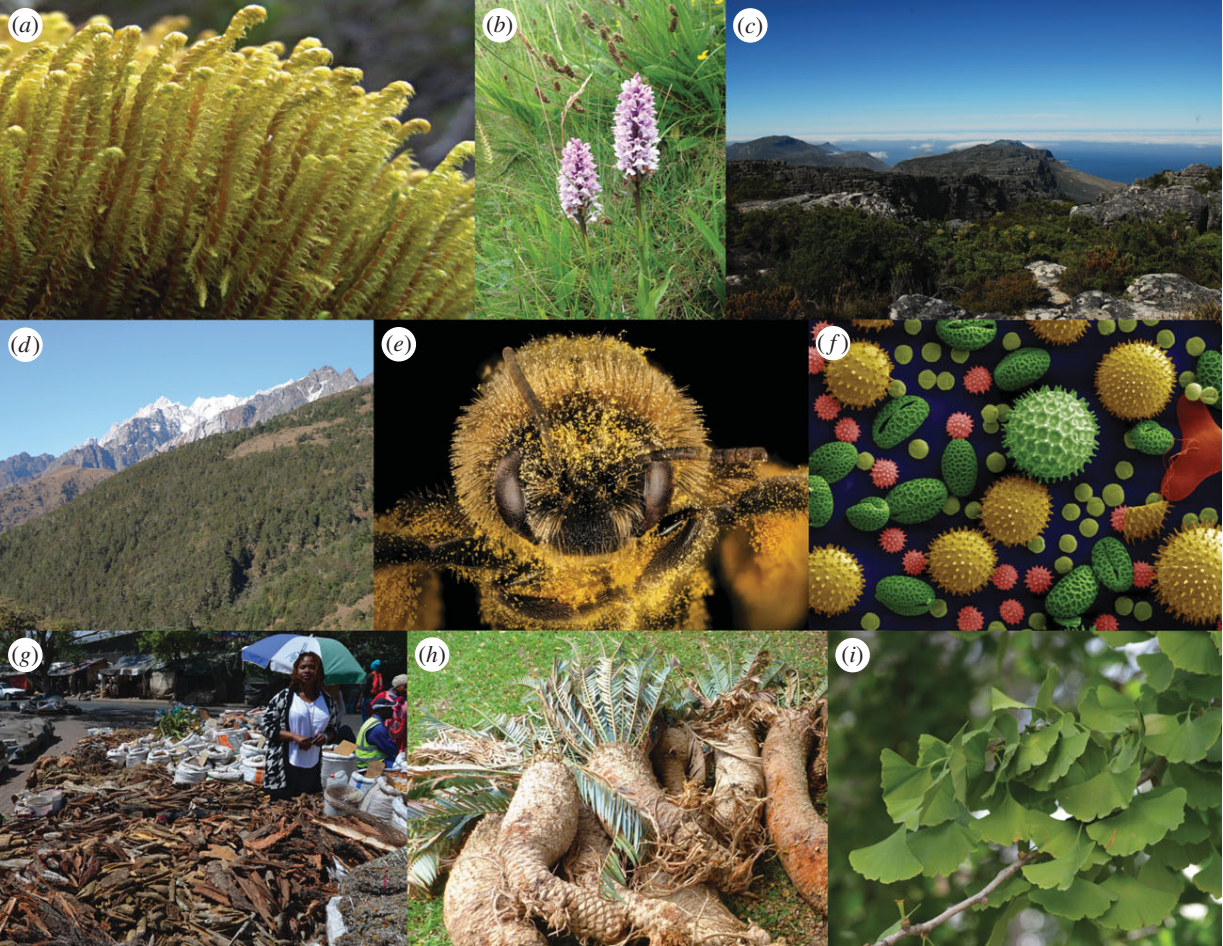
Example uses of DNA barcoding. (*a*) Species discovery in the bryophyte *Herbertus* (Herbertaceae). Image: David Genney, (*b*) first complete national DNA barcode database, for the flora of Wales. Image: Alex Twyford, (*c*) floristic barcoding of the Cape Flora. Image: Olivier Maurin, (*d*) DNA barcoding the flora of China. Image: De-Zhu Li, (*e*) pollen identification and the study of pollen movement. Image: USGS Bee Inventory and Monitoring Lab, (*f*) species identification of historical mixed pollen samples. Image: Dartmouth Electron Microscope Facility, (*g*) a stand selling plant products in Johannesburg. Image: Zandisile Shongwe, (*h*) confiscated illegal *Encephalartos* (Zamiaceae), Image: Eastern Cape Department of Economic Development, Environmental Affairs and Tourism, (*i*) identification of plant compounds (here extract from *Ginkgo biloba*) in herbal supplements. Image: Juan Carlos Lopez Almansa.

Plant barcodes are typically used in an integrative fashion with other information for detecting new taxa. In some studies, unexpected sequence divergence has led to re-examination of morphological/ecological variation, which has then resulted in formal recognition of new species [13]. In other cases, morphological or ecological variants have been the trigger for generating sequence data to establish whether there is supporting genetic evidence for recognizing different taxa [14]. Species discovery has involved the full spectrum of species from relatively small and/or character-poor groups like bryophytes (figure 1*a*) through to conspicuous ecologically/culturally important trees, and in a small number of cases, the nucleotide variants themselves have been formalized into the species descriptions (e.g. [15,16]).

### (b) Vegetation and floristic surveys

Geographically restricted floristic assemblages represent an inherently lower discrimination challenge for plant barcodes, as the closest relatives of many taxa may be absent from the area. Floristic barcoding projects have been completed at a range of scales from plot-level studies of tropical trees [17,18], nature reserves [19,20], local flora [21] and small countries (figure 1*b*, [22]). Ongoing large-scale barcoding projects include steps to complete the barcoding of the flora of South Africa and an ambitious multi-institute project to barcode the flora of China (figure 1*c,d*). Not surprisingly, there is large variation in the percentage of species discriminated (table 1), and this is strongly affected by the geographical scale of study and the complexity of the flora [20,22,24]. Although many other factors are at play, the larger the scale of the study, and the greater the number of species-rich genera, the lower the discrimination success.

**Table 1.** Levels of species discrimination from floristic barcoding studies at different scales and levels of floristic complexity.

study type	study location	no. species	markers	species discrimination (%)	references
tropical trees, forest plot	16-ha plot, northeast Puerto Rico	143	*rbcL*, *matK*, *trnH-psbA*	93	[17]
tropical trees, forest plot	50-ha plot, Cameroon	272	*rbcL*, *matK*, *trnH-psbA*	71–88	[18]
nature reserve	348-ha, Ontario, Canada	436	*rbcL*, *matK*, *trnH-psbA*	95	[19]
nature reserve	1133-ha, Guangdong, China	417	*rbcL*, ITS2	65	[20]
local flora	20 000-ha Churchill, Manitoba, Canada	312	*rbcL*, *matK*, ITS2	69	[21]
national flora	2 m-ha, Wales, UK	1041	*rbcL*, *matK*	69–75	[22]
(large) regional flora	Canadian arctic	490	*rbcL*, *matK*	56	[23]

Moving back in time from contemporary floristic barcoding, several studies have used the *trnL* intron P6 loop to reconstruct historical vegetation types based on environmental sequencing from frozen sediments dating back thousands of years (e.g. [25–27]). Although the small size of the P6 loop (which makes it so well-suited for recovery from ancient samples) inevitably constrains its resolving power at the species level, the approach does provide a standard scalable approach for broad-brush identification, which can increase resolution beyond that of morphological palynology in some plant groups [26].

### (c) Ecological forensics

Floristic barcoding datasets provide a foundation for studies of ecological processes. Conventional identification of plants from individual tissue types/juvenile life stages is usually difficult as the seedlings, roots, seeds and pollen and other gametophytes of many species can appear similar. If the material has been processed in one way or another (e.g. been digested), the difficulties of identification are exacerbated. Thus, as with paleobarcoding, even barcode datasets with imperfect species resolution can still provide knowledge gains. For instance, Kartzinel *et al.* [28] barcoded faecal samples from African herbivores and showed clear dietary niche partitioning even among similar coexisting species. Likewise, Kesanakurti *et al.* [29] used barcode data to show strong spatial structuring of plant roots in the absence of corresponding above-ground structuring. The field of pollen barcoding is growing rapidly, and even modest increases in discriminatory power beyond morphological identification (figure 1*e,f*) hold great promise to enhance understanding of the dynamics and consequences of pollination and pollen movement [30–32].

### (d) Identification to support regulatory enforcement

Reliable identification of plant material by regulatory/enforcement authorities is a widespread need, including identification of pests, pathogens and invasive species to inform control [33,34], detecting protected species being illegally traded (figure 1*g*, [35,36]), through to identifying food or herbal medicine labelling errors/fraud (figure 1*h*, [37]).

Although some applications require species-level resolution, many do not. For instance, useful insights into the composition of food and drink can be obtained at the level of them containing ‘something other than what is on the label’, and Stoeckle *et al.* [38] showed that about one-third of herbal teas contained plant species beyond those listed. Likewise, many studies have deployed DNA barcoding approaches to assess the plant components of herbal medicines and dietary supplements, and evidence of market substitution/adulteration is not uncommon [39–41]. For instance, Little [42] found evidence that 3/37 Ginkgo herbal supplements contained fillers with no detectable Ginkgo DNA (figure 1*i*), and Kumar *et al.* [43] showed evidence for widespread mislabelling of Bala herbal products in market samples.

### (e) DNA barcoding and community phylogenetics

The differing levels of variability among standard plant barcode regions means that commonly deployed markers (e.g. *rbcL, matK, trnH-psbA* and ITS) can provide resolution at different phylogenetic levels, which has facilitated studies of community phylogenies [17,44], comparative biology and phylogenetic diversity. Shapcott *et al.* [45] used plastid barcodes to identify priority areas for conservation in Australian rainforests based on both species richness and phylogenetic diversity (involving the identification of areas containing more phylogenetic diversity than would be expected based on species richness alone). Using floristic phylogenies in a rather different manner, Saslis-Lagoudakis *et al.* [46] capitalized on barcode datasets for the floras of the Cape of South Africa, Nepal and New Zealand to study the phylogenetic distribution of plants used in traditional medicine. They showed significant phylogenetic clustering of traditionally used medicinal species and highlighted cases where different cultures have exploited the same lineages for bioactive compounds, and noted the predictive capacity of the phylogenies for further screening for bioactives.

## 3. Limitations of standard plant barcodes

Pilot studies, careful project design and an appropriate match of inference to the level of signal in the data are critical to the effective use of standard plant barcodes, and these principles underpin many of the studies described above. This is necessary as the literature is replete with examples of plants sharing barcodes among related species and numerous cases where uniquely distinguishable species in a genus are the exception not the rule [12]. Thus, uncritical use of plant barcoding may lead to disappointing and/or uninformative results. Beyond this fundamental challenge of restricted/variable discriminatory power, there are additional practical issues such as primer mismatches impacting on the recovery of *matK* barcodes, as well as ongoing different preferences for different barcode regions for different applications that make it difficult to combine reference datasets generated for different purposes or studies [12].

## 4. Factors influencing the discriminatory power of standard plant barcodes

Various studies have been undertaken to unpick the reasons why plant barcodes are often shared between related species. Obvious drivers include hybridization, groups with slow mutation rates relative to speciation rate, and general challenges in groups showing recent and rapid divergence [12,24,47,48]. Somewhat less obvious is the notion that the limited seed dispersal compared with pollen dispersal in many plant species may act as an intrinsic limitation on the degree to which maternally inherited plastid barcodes are likely to track species boundaries [12,49]. This is attributable to the low intra-specific gene flow of seed-dispersed plastid markers essentially retarding the ability of new variants to spread throughout a species range, and a related increase in the likelihood of successful local interspecific introgression [12,50]. Likewise, selective sweeps acting on the plastid genome combined with hybridization have also been invoked in limiting the resolving power of plastid barcodes—best exemplified by the remarkable case of *Salix*, where 337 individuals from 53 species from 3/5 subgenera across Europe and North America share a barcode haplotype [51,52]. The use of nuclear ITS often increases levels of resolution beyond those of plastid markers but within limits [5,6]. In some groups, multiple copies occur, creating challenges of sequencing and/or interpretation of paralogues, and interspecific barcode sharing either through lack of divergence or hybridization is also not uncommon in ITS datasets [5].

Plant barcoding is at something of a crossroads. On the one hand, there are a multitude of applications that are well suited for the existing resolving power of plant barcodes and continuing these studies, and establishing sample sets to support the reference databases remains a high priority and focus for the plant-barcoding community. On the other hand, given the limitations of discriminatory power of standard barcodes in many plant groups, there is a clear and unambiguous need for improved barcoding protocols.

## 5. Extending and improving the plant barcode

### (a) Additional amplicon sequencing

One option to improve species discrimination in plants is supplementing standard plant DNA barcodes with additional loci generated with Sanger Sequencing or via NGS of tagged amplicons [53]. The benefits here are that Sanger Sequencing is inexpensive on a per-individual basis, and that there have been methodological improvements in generating these data (e.g. improved DNA polymerases). While there are some candidates and improvements in discrimination for individual groups [53,54], most evidence suggests that the gain in species discrimination will be incremental [55,56]. This is particularly the case as barcoding with Sanger Sequencing is limited to organellar and ribosomal loci, as cloning heterozygous nuclear loci is not feasible. Even where Sanger Sequencing gives way to NGS of barcoded amplicons, these approaches are typically constrained to a small set of nuclear loci, and evidence to date suggests sometimes very modest discrimination gains from sequencing ∼ 10 nuclear regions as barcodes due to lack of intraspecific coalescence [57,58].

### (b) Plastid genome sequences

Several authors have argued for having complete plastid gene sets, or indeed complete plastid genomes, as the plant barcode [59,60]. The highly conserved gene order, the absence of recombination and low levels of nucleotide substitution make the plastid the ideal target for comparative analysis across the land plants. In addition, the high-copy number means genomic DNA extracts are enriched for plastids, and thus an easier target than low-copy nuclear genes for sequencing, particularly from degraded samples. Complete (or near-complete) plastid genomes can be obtained by short- or long-ranged PCR enrichment with conserved primers [61,62], direct isolation protocols [63], capture via oligonucleotide probes [64] or genome skimming of genomic DNA [65].

Sequencing the complete plastid genome provides more characters and increases the amount of sequence data by two orders of magnitude (e.g. from approx. 1400 bp for *rbcL* and *matK* to approx. 150 000 bp), and this can provide some increase in species discrimination (e.g. [58]). Use of complete plastid genomes also gets around the problem of different research groups favouring different plastid regions, as the reference database essentially covers all plastid barcodes [66]. Complete plastid genome sequencing fits the requirement of being highly scalable, with reliable automated assemblers (e.g. ORG.asm assembler [67]), annotation [68] and broad-scale alignment [69] possible for all but the most structurally divergent land-plant plastid genomes such as those found in parasitic, mycoheterotrophic or carnivorous taxa [70].

However, complete plastid sequencing does not address the basic challenge that plastid genomes do not necessarily track species boundaries [12,71,72]. Thus, although we envisage the coming few years will see a steep increase in the number of publications using complete plastid genomes as barcodes, the ultimate big gains in resolving power will only come with access to substantial numbers of unlinked nuclear markers. There are two obvious primary routes to do this: target enrichment or genome skimming, with additional possibilities including transcriptome sequencing and RAD-seq.

### (c) Targeted enrichment

**Figure 2.**
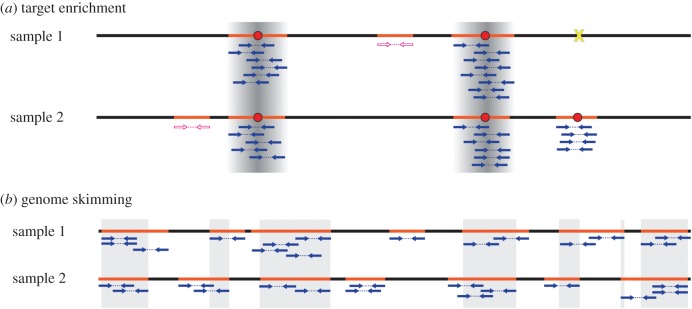
Comparison between promising genomic barcodes. (*a*) Target enrichment focuses sequencing reads (blue arrows) on homologous regions of the genome surrounding bait sites (red dots), with many regions with high coverage (dark-grey shading). Samples missing a suitable bait site (yellow cross) are not represented in the data. Off-bait reads (pink open arrows) may be informative, particularly if they map to high-copy ribosomal DNA or organelles. (*b*) Genome skimming can be used to generate a fragmented nuclear assembly with low sequence coverage. Homologous sequences are a random collection of regions where assemblies overlap (grey boxes).

Targeted enrichment includes approaches that use short oligonucleotide probes (baits) to pull down homologous sequences in a genomic DNA extract, with the enriched DNA then subject to NGS (figure 2*a*, [73]). The approach is highly scalable and well suited for recovery from degraded DNAs [73,74]. The key question here is whether a universal probe set can be developed to capture a large set of homologous loci across all land plants [66].

While it is clear that there are nuclear loci conserved across large groups of plants, such as the 1025 conserved orthologue set loci between tomato and *Arabidopsis* [75], or the 1083 putative orthologues in the genomes of seven angiosperms and a moss species [76], there is no single set of well-curated nuclear genes. However, there are a wealth of resources that could be used to find conserved loci, particularly transcriptomes from the oneKP project ([77] onekp.com), or the 58 complete plant genomes (https://phytozome.jgi.doe.gov). A conserved set of baits designed from these resources could be supplemented with available baits for large and important clades, such as the Compositae [78]. As such an important issue is the balance between low-variation universal loci, or more variable loci that can only be retrieved from a subsample of species.

Going from a candidate gene set to an effective hybridization assay is non-trivial. Early studies showed that most conserved *Arabidopsis* loci do not hybridize to tomato baits under stringent conditions [75], probably due to sequence divergence. On the other hand, using lower stringency conditions will capture off-target sequences and paralogues, which will affect downstream applications. Here, the challenge is designing short probes that can effectively bait a single specific target locus. A landmark study in the application of baits to phylogenetically disparate taxa [64] used a suite of 55 000 RNA baits, each 120 bp in length, to capture entire plastids. The short probe length used here would be particularly useful for capturing loci from degraded samples. However, it is unclear whether such a phylogenetically diverse range of land plants could be assayed with a single nuclear gene set. This is a high-priority area for assay development.

### (d) Genome skimming

A genomic DNA extract typically contains a mix of nuclear and organellar DNA (plastid and mitochondria), and NGS will generate data across the three genomes. At low sequence coverage (e.g. 0.1–10×, approx. 1 GB of data), the genome can be ‘skimmed’ [65], allowing the near-complete assembly of the high-copy plastid, mitochondria and ribosomal RNA (figure 2*b*). There is also the potential to make a highly fragmented nuclear genome assembly.

Genome skimming has great promise for extending the plant barcode, reviewed by Coissac *et al.* [66]. Importantly, genome skimming is scalable and (relatively) cost-effective, and can be used effectively with degraded DNAs from herbarium specimens [79]. At the lower end, benchtop protocols for single insert–size library preparation, such as the Illumina Nextera and TruSeq, can be performed on a small number of samples. For larger applications, library preparation can be automated on robotic liquid handlers such as the Illumina NeoPrep. These libraries can then be multiplexed on a range of sequencing platforms, with the cheapest per-sample-costs with high-output platforms (box 1). Downstream, parts of the data assembly are suited to automation, particularly organelle assembly (e.g. plastids [67,79], mitochondria [85]). In terms of costs, library preparation and low-coverage sequencing can be $200 per sample when highly multiplexed [66].

Box 1. **Recent developments in NGS platforms.**

*Increased output*. The Illumina HiSeq X and HiSeq 4000 sequencers use patterned flow cell technology to generate extremely high output. The HiSeq 4000 generates 750 GB data per run, enough to sequence 90 *Arabidopsis* genomes at 60× coverage. These platforms will greatly reduce the cost of projects that use a large number of short reads (up to 150 bp paired-end), such as genome skims.

*Longer read lengths*. Current long-read sequencers include the Pacific BioSciences real-time sequencer [80] and Oxford NanoPore's MinION [81]. These PCR-free single molecular sequencing platforms generate reads many kilobases in length (PacBio > 10 kb, MinION > 5 kb), with these data widely used to scaffold genomes assembled from inexpensive Illumina data [82]. Their immediate use for barcoding is unclear due to their high error rates and sequencing costs, though proof-of-concept studies suggest that these platforms are promising [83].

*Portable sequencers*. Oxford NanoPore's MinION is the first portable NGS platform. This pocket-sized device allows sequencing to be done anywhere, only requiring a connection to a laptop. Other benefits include the low lease cost and the production of data in real time. Portable genomics has great potential and may enable barcoding in the field. While field-based sequencing has become reality for studying the spread of viruses [84], for field barcoding of plants there will need to be new sample assays that focus the modest sequencing output onto homologous regions.

*In-house genomics*. The high purchase costs and the requirement for specialized laboratory skills have limited NGS platforms to large centralized sequencing hubs. This is likely to change with the release of low-output sequencing platforms intended for small research groups. The most prominent is the Illumina MiniSeq, which costs $50 000, has a small footprint, and produces 7.5 GB of data overnight. This platform could be extremely useful for barcoding work with amplicons or enriched samples, such as those from hybrid baits. It could also be used for preliminary genomics of challenging samples such as those from degraded tissues.

A second benefit of genome skimming is that it is both backwards-compatible with the standard plant barcodes, and forwards-compatible with genome sequencing (discussed below) [66]. Genome skims routinely recover plastid barcode loci and ITS, and thus continue to add to the growing reference database of the standard barcoding loci. In terms of compatibility with future genome sequencing approaches, the archived sequence reads that can be reassembled as improved assembly algorithms become available, while archived DNA samples or NGS libraries could be resequenced to provide better coverages as costs decrease [66].

A significant challenge for using genome skimming for DNA barcoding is how to effectively use the nuclear data. Many genome-skimming studies discount the nuclear reads and only assemble the organellar and ribosomal DNA [65,86,87]. While nuclear assemblies are possible using assemblers intended for large diploid genomes (reviewed in [79]), the combined factors of low sequence coverage, short-read lengths and single small DNA insert size means the nuclear assembly will be a near-random collection of fragmented DNA sequences. An assembly from a single-insert library will often have a median contiguous DNA size (N50) of around 5–10 kb, with the largest fragments in the range of 30–120 kb in length (AD Twyford, 2016 unpublished data). To use this for species discrimination will rely on algorithms that can cope with comparisons among sample sets with highly variable and patchy overlap in the data [66].

### (e) Other technologies

One of the most popular approaches to access large numbers of nuclear markers is through the sequencing of regions adjacent to restriction-enzyme cut sites, including genotyping by sequencing [88] and restriction site–associated DNA sequencing (RAD [89,90]). These approaches allow thousands of homologous regions to be sequenced across hundreds of individuals, without prior knowledge of the genome sequence. While there are cases where these methods have been informative across species clades (e.g. [91–93]), the lack of conserved cut sites across a very broad taxonomic scope makes them better suited to closely related taxa. While RAD has its benefits, and deserves more thorough testing for species discrimination in individual clades, we do not see this as a primary route for universal barcoding.

Transcriptome sequencing is a widely used tool for the analysis of gene expression, marker discovery and comparative evolution [94]. The main benefit of transcriptomics is that it focuses NGS onto a homologous proportion of the genome, which in this case is also the most highly conserved. However, the requirement for high-quality fresh material, and the tissue-specific nature of the sequences, rules it out for universal barcoding.

### (f) Entire genomes

The gold standard in genome sequencing are model organisms such as *Drosophila*, *Arabidopsis* and humans, where sequence reads mapped to high-quality reference genomes allow chromosome-level assemblies encompassing most of the genome [95]. There are also many cases where high-quality reference genomes have been assembled *de novo* from diverse wild organisms [96,97]. While many plant genomes are now publically available, there are major technical and biological hurdles to making whole-genome sequencing scalable and cost-effective. The biggest limitation to *de novo* plant genome assembly are repetitive sequences and the associated large variation and size of plant genomes (plants vary 2000-fold in their genome sizes [98], with a number of groups containing species with giant genomes, e.g. more than 40 GB in *Fritillaria*, [99]). And although there are many other reasons why huge datasets of complete genome sequences are highly desirable, for the particular challenge of species discrimination there would be substantial redundancy in the data.

## 6. Concluding remarks

In this paper, we have outlined the strengths and diverse applications of standard plant barcodes but also noted their limitations. We have summarized some of the exciting future directions made possible by developments in sequencing technologies. However, it is important to qualify this future enthusiasm with a healthy dose of pragmatism. DNA barcoding involves huge sample sets [10]. Part of its success has been based around industrial-scale thinking of laboratory practices and informatics pipelines. The challenges of data editing, quality checking, analysis and storage for standard barcodes are far from trivial, and massively upscaling the depth of data per individual is a huge undertaking. Likewise, although NGS costs continue to fall, the per-sample library preparation costs are still prohibitive in many cases. Large-scale projects involving thousands of samples are underway using genome skimming [66], and the informatics pipelines are progressing rapidly. There are, however, considerable developments and cost reductions required before ‘Plant Barcoding 2.0’ can be considered truly scaleable and widely adoptable, especially to less well–resourced laboratories. With this in mind, we advocate a twin-track approach of (i) continued construction of the reference library via large-scale sample sets and careful deployment of standard plant barcodes, while (ii) maintaining and enhancing international collaborative efforts to further develop plant barcode protocols to support the ultimate objective of establishing a workflow with the resolving power to uniquely discriminate the vast majority of the world's land plant species.
